# Envelope statistics of self-motion signals experienced by human subjects during everyday activities: Implications for vestibular processing

**DOI:** 10.1371/journal.pone.0178664

**Published:** 2017-06-02

**Authors:** Jérome Carriot, Mohsen Jamali, Kathleen E. Cullen, Maurice J. Chacron

**Affiliations:** Department of Physiology, McGill University, Montreal, Québec, Canada; University of Muenster, GERMANY

## Abstract

There is accumulating evidence that the brain’s neural coding strategies are constrained by natural stimulus statistics. Here we investigated the statistics of the time varying envelope (i.e. a second-order stimulus attribute that is related to variance) of rotational and translational self-motion signals experienced by human subjects during everyday activities. We found that envelopes can reach large values across all six motion dimensions (~450 deg/s for rotations and ~4 G for translations). Unlike results obtained in other sensory modalities, the spectral power of envelope signals decreased slowly for low (< 2 Hz) and more sharply for high (>2 Hz) temporal frequencies and thus was not well-fit by a power law. We next compared the spectral properties of envelope signals resulting from active and passive self-motion, as well as those resulting from signals obtained when the subject is absent (i.e. external stimuli). Our data suggest that different mechanisms underlie deviation from scale invariance in rotational and translational self-motion envelopes. Specifically, active self-motion and filtering by the human body cause deviation from scale invariance primarily for translational and rotational envelope signals, respectively. Finally, we used well-established models in order to predict the responses of peripheral vestibular afferents to natural envelope stimuli. We found that irregular afferents responded more strongly to envelopes than their regular counterparts. Our findings have important consequences for understanding the coding strategies used by the vestibular system to process natural second-order self-motion signals.

## Introduction

Understanding the set of transformations by which sensory input gives rise to behavior (i.e. the neural code) remains a central problem in systems neuroscience. Growing evidence suggests that the coding strategies used by sensory systems are adapted to the statistics of natural input [1–9], thus making knowledge of these statistics vital for understanding the neural code. The prevailing view is that natural stimuli display scale invariance (i.e., they are self-similar when observed at different temporal or spatial scales). As a result, their spectral power decays as a power law with increasing spatial or temporal frequency [3]. Studies performed across systems have shown that the properties of sensory neurons optimize their coding of natural stimuli based on both probability of occurrence in the natural environment [1] as well as their spectral structure. For the latter, optimized coding can be achieved by decorrelating the sensory input: such “temporal whitening” has been observed across systems and species and requires that a neuron’s tuning curve opposes stimulus spectral power such that the neural response to natural stimulation is independent of frequency (i.e., “white”) [4–6, 8, 10, 11].

In the temporal domain, natural stimuli frequently consist of a fast time-varying waveform (a first-order attribute that is also referred to as the carrier) whose amplitude (i.e. a second-order attribute commonly referred to as the envelope) varies independently and more slowly [3, 12–18]. There is accumulating evidence that envelope waveforms carry critical information and thus must be encoded by the brain [14, 19–24]. Notably, as the envelope temporal frequency content differs from that of the carrier, recovering the envelope of a signal (also known as signal demodulation) can only be achieved by nonlinear transformations [19, 25]. This fundamental property required for the demodulation of envelopes complicates efforts to understand how these behaviorally relevant stimulus features are encoded in the brain.

To this end, we took advantage of the vestibular system which is well-defined anatomically and physiologically and benefits from easily characterized sensory stimuli (i.e., head acceleration/velocity). The vestibular system is essential for the generation of the most automatic reflexes, as well as for accurate spatial perception and motor control [26, 27]. Vestibular afferents innervate the receptor cells of the vestibular sensors and provide crucial information about head motion to target neurons in the central vestibular nuclei. In the absence of stimulation, vestibular afferents display a wide range of resting discharge variability and are characterized as regular or irregular- a classification that correlates with differences in morphological features and response dynamics [28–31]. Both afferent classes in turn project to reflex pathways as well as higher brain areas, thereby mediating perception and behavior.

To date, the responses of afferent and their central vestibular neural targets have been almost exclusively characterized using artificial (e.g. sinusoidal, noise) stimuli, leading to the conventional wisdom that early vestibular processing is inherently linear [28, 32, 33]. If this were the case, then single vestibular neurons should not respond to the time varying envelope of self-motion signals. However, recent studies have shown that vestibular neurons respond nonlinearly to naturalistic self-motion stimuli [34, 35] and thus actually respond to envelopes [36]. Furthermore, the encoding of envelopes by the vestibular system may be important for adapting sensory processing to the current stimulus amplitude range, as has been observed behaviorally [37–39]. Recent studies have characterized the statistics of carrier self-motion signals [40] and shown that the tuning of peripheral afferents is adapted to optimally encode these [34]. However, whether vestibular pathways have also adapted to optimally encode natural second-order self-motion signals based on ther statistics is unknown, in part because these statistics have not been characterized to date.

## Methods

### Ethics statement

Informed written consent was obtained from all subjects before the study. All experiments and procedures including obtaining informed written consent from all subjects were approved by McGill University’s Human Ethics Committee. All experiments were furthermore performed in accordance with the guidelines of Ethical Conduct for Research Involving Humans. All data were gathered and previously analyzed for first-order self-motion signal statistics in [40].

### Subjects and head movement recordings

Head movements were recorded in 8 healthy human subjects with no past history of visual or vestibular impairments (4 male, 4 female; age, 22–34 years) during normal everyday activities. We used a micro-electromechanical systems (MEMS) module (**i**NEMO platform, STEVAL-MKI062V2, STMicroelectronics) that combined three linear accelerometers (linear accelerations along the Fore-Aft, Inter-Aural, and Vertical axes) and was augmented by a STEVAL-MKI107V2 three axis gyroscope (angular velocity about pitch, roll, and yaw). Data from all six sensors were sampled at 100 Hz and recorded on a microSD card. All equipment (MEMS module, battery, microSD card) were positioned on a small light enclosure that could be comfortably attached to the subject’s head or fixed to the environment (e.g., a seat in a vehicle). The Fore-Aft and Inter-Aural axes were set parallel to the subject’s Frankfurt plane (i.e., the plane passing through the inferior margin of the orbit and the upper margin of the external auditory meatus), as done previously [40]. The noise level in the MEMS module was determined by recording signals for 15 minutes while not moving.

### Activities

Each subject was asked to perform everyday activities each typically lasting 2 minutes in a random order. Activities consisted either of voluntary (i.e. active) (walking, going up and down the stairs, running, running through the woods, sprinting, jumping forward, jumping up and down, hopping on one foot, playing soccer, biking on a city street, biking on a grassy field) or passively applied (riding the city subway seated, riding the city subway standing up, riding a city bus seated and, riding a city bus standing up) self-motion.

### Data analysis

Recorded angular velocity signals were projected onto the semicircular canal planes (left anterior–right posterior [LARP], right anterior–left posterior [RALP], and YAW) as done previously [40]. The signals obtained for different activities (i.e., passive, active, or both) were then concatenated for each subject as done previously [40]. We note that this approach is similar to that used in other systems [41–43]. The time varying amplitude or envelope *E(t)* was then extracted from each resulting signal *S(t)* (i.e. either angular velocity along the YAW, LARP, and RALP axes; or linear acceleration along the Fore-Aft, Inter-Aural, and Vertical axes) using the Hilbert transform [21, 36, 44, 45]:

$$E\left(t\right)=\sqrt{S{\left(t\right)}^{2}+X{\left(t\right)}^{2}}$$

$$X\left(t\right)=\frac{1}{\pi }C\left[\int_{-\infty }^{+\infty }\frac{S\left(\tau \right)}{t-\tau }d\tau \right]$$

where C[…] is the Cauchy principal value. Probability distributions were obtained using binwidths of 0.01 G and 10 deg/s for linear acceleration and angular velocity, respectively. Since the envelope can only be positive by definition, we fitted a half-Gaussian to the probability distribution. The excess kurtosis was then computed as:

$$K=\frac{\langle {\left(x-\mu \right)}^{4}\rangle }{\sigma^{4}}-3$$

where *μ* and *σ* are the mean and standard deviation of the distribution, respectively.

Power spectral densities of the signals recorded during self-motion were computed using Welch’s average periodogram with 512 points and a Bartlett window (512 ms duration).

Power spectral densities were fit using a single power law model (model 1):

$$P_{1}\left(f\right)=\frac{A}{f^{\alpha }},$$

where A is a constant, f is frequency, P is power, and α is the power law exponent. In practice, parameter values were obtained by performing a linear least squares fit on the logarithms of the power spectral density and frequency over the range 0–50 Hz.

We also used a double power law model (model 2):

$$P_{2}\left(f\right)=\{\begin{array}{cc} \frac{A_{1}}{f^{\alpha_{1}}} & if\;f\leq f_{t}\\ \frac{A_{2}}{f^{\alpha_{2}}} & if\;f>f_{t} \end{array}$$

Here *A*_*1*_, *A*_*2*_ are constants, *α*_*1*_, *α*_*2*_ are the power law exponents, and f_*t*_ is the transition frequency. All parameter values were obtained by performing a linear least squares fit on the logarithms of the power spectral density and frequency over the ranges 0-f_*t*_ and f_*t*_-50 Hz.

The cutoff frequency f_*t*_ was determined in the following way. The goodness of fit of each model was assessed by computing the variance-accounted-for given by:

$$VAF=1-\frac{VAR\left(y-\hat{y}\right)}{VAR\left(y-\bar{y}\right)}$$

where VAR(…) is the variance, y is the data, $\hat{y}$ is the fit to the data, and $\bar{y}$ is the mean of the data. The sampling interval of the data increased exponentially, such that the datapoints were evenly spaced when taking the logarithms of power spectral density and frequency. This was done in order to give equivalent weighting for low and high values of the logarithm of frequency. The transition frequency was chosen as that for which the VAF was maximized.

We determined which model was the best fit to the data by testing whether the low frequency power law exponent was significantly different than the high frequency power law exponent when using model 2. If this difference was not significantly different from zero, we used model 1 to fit the data. Otherwise, if the difference was significantly different from zero, then we used model 2 to fit the data.

### Statistics

Values are reported as mean ± STD throughout unless otherwise noted. The shaded gray bands in the figures show 1 STD.

### Modeling

We first used previously established linear models to predict afferent responses to the experimentally recorded natural stimuli. Specifically, we assumed that the output firing rate *r(t)* in response to stimulus *S(t)* is given by the following: *r*(*t*) = *r*_0_ + (*H* * *s*)(*t*), where the asterisk denotes a convolution with a filter *H(t)* and *r*_*0*_ is the baseline (i.e., in the absence of stimulation) firing rate. We used *r*_*0*_ = 100 Hz which corresponds to the average baseline firing rate observed experimentally [46]. We used standard expressions for the Fourier transform of *H(t)* (i.e., the transfer function) [34, 35]:

$$\tilde{H}\left(f\right)=k\frac{2\pi if\left(2\pi if+1/T_{1}\right)}{\left(2\pi if+1/T_{c}\right)\left(2\pi if+1/T_{2}\right)},$$

where *f* is frequency and $i=\sqrt{-1}$. For regular afferents, we used *k* = 2.83 (spk/sec)/(deg/sec), *T*_*1*_ = 0.0175 s, *T*_*2*_ = 0.0027 s, and *T*_*c*_ = 5.7 s. For irregular afferents, we used *k* = 27.09 (spk/sec)/(deg/sec), *T*_*1*_ = 0.03 s, *T*_*2*_ = 0.0006 s, and *T*_*c*_ = 5.7 s. The output firing rate was then passed through a clipping nonlinearity: rectification was implemented by setting negative values of *r(t)* to zero while saturation was implemented by setting values of *r(t)* greater than 400 spk/sec to 400 spk/sec. These values were taken based on experimental observations [34, 46] and the value used for saturation did not qualitatively affect the nature of our results (not shown).

Finally, the sensitivity to the envelope was computed as:

$$G\left(f\right)=\frac{P_{er}\left(f\right)}{P_{ee}\left(f\right)},$$

where *P*_*er*_*(f)* is the cross-spectrum between the output firing rate after implementing the clipping nonlinearity and the envelope, and *P*_*ee*_*(f)* is the power spectrum of the envelope. We note that the sensitivity is the ratio of the output to the input amplitude at a given frequency and has been previously used to quantify tuning to envelopes in the electrosensory system [8, 10, 21, 47] as well as in the auditory system [48, 49] (see [15] for review).

## Results

### Envelope statistics of vestibular signals during natural self-motion

**Fig 1:**
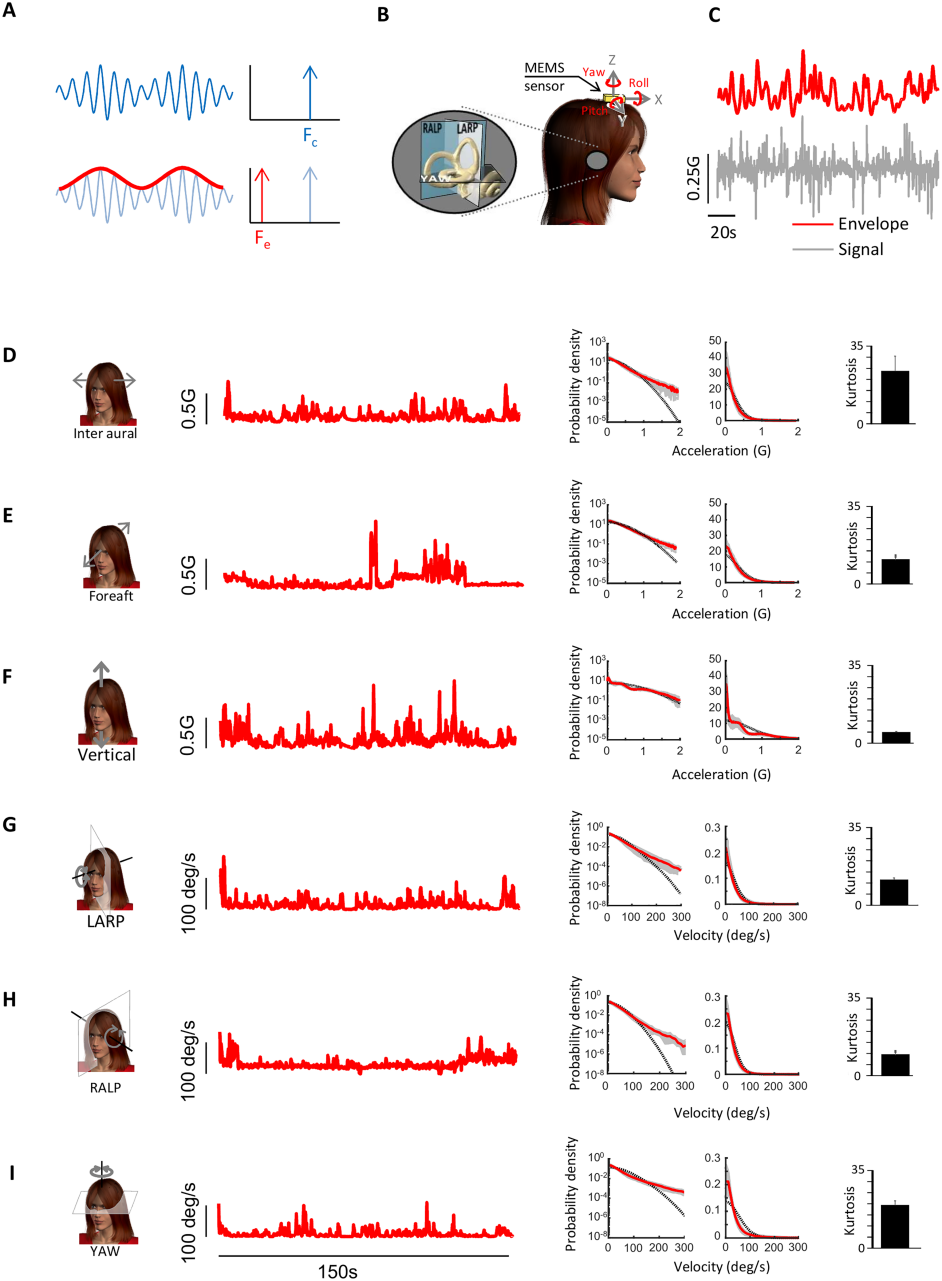
Envelope statistics of self-motion signals experienced during everyday activities. **A:** Schematic showing a sinusoidal trace whose amplitude also varies sinusoidally (blue trace, top left). The power spectrum is non-zero only at the carrier frequency F_c_ (top right). The envelope of the signal (red trace, bottom left) oscillates with a different frequency F_e_ than that of the full signal as confirmed by taking its power spectrum (bottom right). **B:** A MEMS module consisting of three gyroscopes and three linear accelerometers was mounted on the subject’s head and measured linear accelerations along the Fore-Aft, Inter-Aural and Vertical axis as well as rotations about the Pitch, Yaw, and Roll axes. **C:** Example signal (gray) recorded from the MEMS module and its time varying envelope (red). **D,E,F,G,H,I:** Example angular velocity or linear acceleration envelope signals recorded during everyday activities for Inter-Aural (**D**), Fore-Aft (**E)**, Vertical (**F**), LARP (**G**), RALP (**H**), and YAW (**I**). In each case, shown are an example time series (left), the probability distributions plotted using logarithmic (middle left) and linear (middle right) scales, together with a Gaussian fit (dashed black), and the population-averaged excess Kurtosis (right). Gray bands show 1 STD.

As mentioned above, it is important to note that the envelope signal can only be extracted by a nonlinear transformation. For example, consider the waveform shown in Fig 1A consisting of a sinusoidal carrier whose amplitude (i.e. envelope) is also varying sinusoidally at a lower frequency. Fourier analysis (which is a linear transformation) performed on the complete waveform reveals that power is present only at the high frequency content of the carrier but not at the low frequency content of the envelope (Fig 1A, top). Thus, to extract the envelope, nonlinear transformations (e.g., half or full-wave rectification) are necessary (Fig 1A, bottom).

In a previous analysis we characterized the first-order statistics of self-motion stimuli (i.e., also referred to as the carrier) experienced by human subjects during natural everyday behaviors (e.g. running, jumping, riding in a vehicle) [40]. Stimuli along six axes of translational and rotational motion were measured using a portable MEMS module that was attached to the subject’s head (Fig 1B). Angular velocity signals were projected onto each subject’s semicircular canal planes (LARP, RALP, and YAW) prior to analysis. Here we instead characterized the second-order statistics of self-motion stimuli (also referred to as the envelope) by applying a nonlinear transformation (see Methods) as done previously for other sensory modalities [44, 45, 50] (Fig 1C).

**Fig 2:**
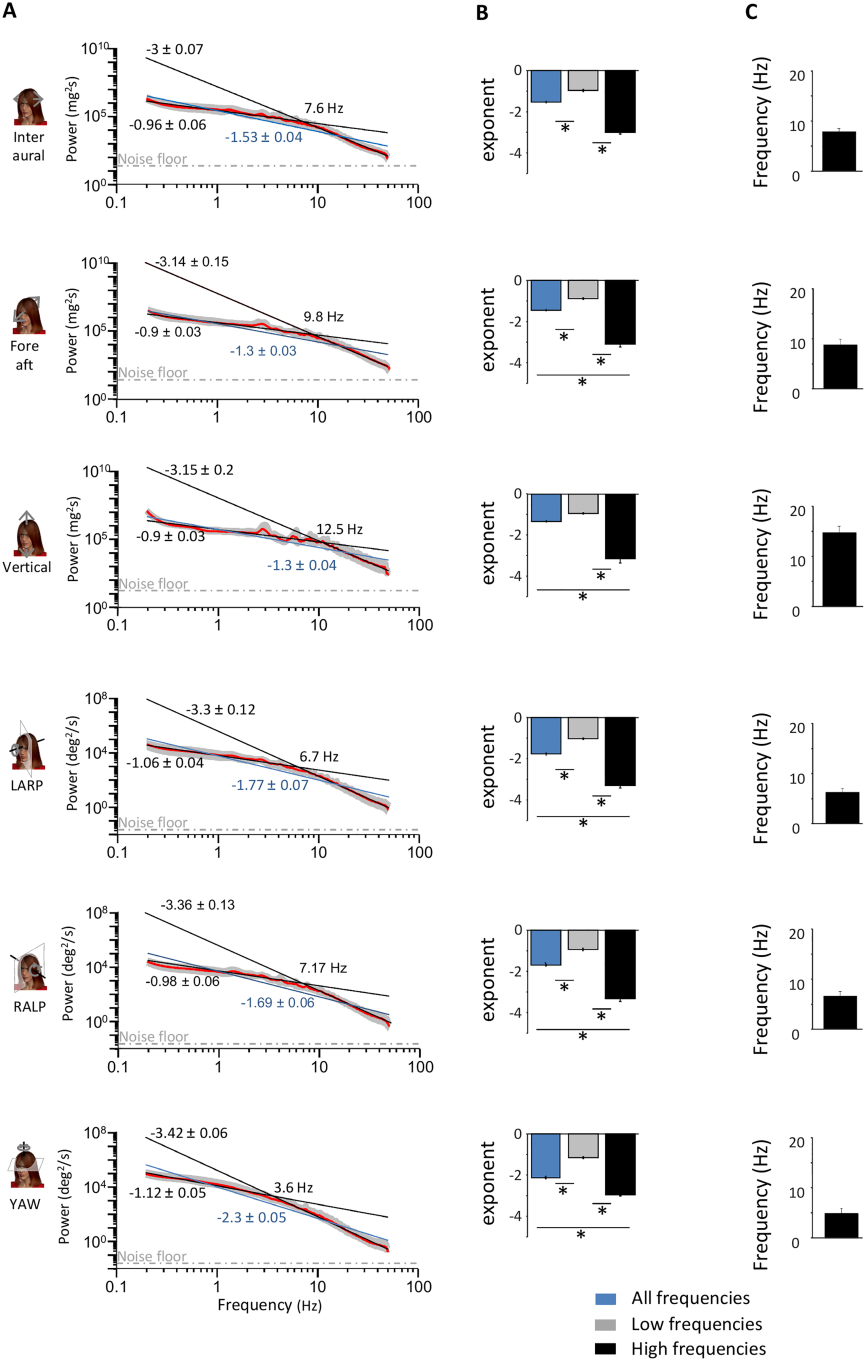
Envelope signals deviate from scale invariance. **A:** Subject-averaged power spectra (red lines) with best-fit power laws over the low and high frequency ranges (black lines) as well as best-fit single power law over the entire frequency range (blue lines). Also shown are the best-fit power law exponents with confidence interval as well as the transition frequency. The dashed gray lines show the “noise floor”, which is the spectrum of the noise in the measurement obtained when the sensor was not moving (see Methods). Gray bands show 1 STD. **B:** Subject-averaged best-fit power law exponents over the low (gray) and high (black) frequency ranges for all six motion dimensions. Also shown for comparison are the subject-averaged best-fit power law exponents for a single power law over the entire frequency range (blue). “*” indicates statistical significance at the p = 0.01 level using a one-way ANOVA. **C:** Subject-averaged frequency at which the power spectrum starts decreasing more sharply for all six motion dimensions.

The envelope statistics of vestibular signals for all activities are summarized in Table 1 (passive) and Table 2 (active). Overall, these signals could reach high values (~450deg/s and ~4G) that varied greatly across activities. Envelope signals were characterized by probability distributions with long tails that decreased more slowly than a half-Gaussian distribution as quantified by large excess kurtosis values (Fig 1D, 1E, 1F, 1G, 1H and 1I). Previous studies performed in other systems have shown that envelope signals are scale invariant (i.e., look similar at different spatial and temporal timescales) [18, 20, 51]. A characteristic of scale invariance is that spectral power will decrease as a power law as a function of frequency. Thus, to test whether the envelopes of natural self-motion signals were scale invariant, we computed their power spectra as a function of temporal frequency. We found that these spectra decreased more sharply for high (> 2 Hz) than for low (<2 Hz) frequencies (Fig 2A) and were thus not well fit by a single power law (blue lines), indicating deviation from scale invariance. The spectra were however better fit by two power laws with exponents near -1 and -3 over the low and high frequency ranges (black lines), respectively (Fig 2A and 2B). The population-averaged best-fit exponents over the low and high frequency ranges were significantly different from one another, and were furthermore significantly different from the best-fit exponent of the single power law model for all six motion dimensions (p<0.01 in all cases, one-way ANOVAs, Fig 2B). The frequency at which the transition from a slow to a fast decrease occurred ranged between 4 and 15 Hz across motion dimensions (Fig 2C). Thus we conclude that the envelopes of vestibular signals encountered across everyday activities that include both active and passive self-motion are not scale invariant prior to reaching the sensory organs in the subject’s head. This result has important consequences for neural coding as further discussed below.

**Table 1:** Subject-averaged maximum value, mean, and kurtosis for passive everyday activities. The maximum and mean values are expressed in mG for the Lateral, Fore-Aft and Vertical linear acceleration while they are expressed in deg/s for the LARP, RALP and Yaw angular velocity.

		Maximum value	Mean	Kurtosis
Bus ride	Inter-Aural	**596.45**±212.30	**135.19**±41.43	**5.11**±1.99
	Fore-Aft	**906.59**±283.24	**172.39**±40.74	**7.32**±5.09
	Vertical	**568.24**±209.38	**78.32**±29.78	**10.81**±3.99
	LARP	**108.96**±35.21	**15.94**±5.54	**11.99**±8.05
	RALP	**89.72**±34.15	**13.09**±5.45	**12.50**±7.43
	Yaw	**230.61**±80.50	**25.91**±12.66	**18.09**±10.47
Car ride	Inter-Aural	**840.40**±200.50	**104.72**±25.54	**11.54**±2.02
	Fore-Aft	**1062.51**±329.70	**141.87** ±19.03	**9.70**±7.95
	Vertical	**555.24**±189.20	**48.17**±6.89	**16.13**±6.57
	LARP	**125.57**±32.51	**12.32**±2.94	**15.56**±7.48
	RALP	**120.89**±20.79	**11.16**±2.75	**21.79**±18.16
	Yaw	**334.02**±76.52	**18.97**±6.12	**34.09**±16.65
Metro ride	Inter-Aural	**506.93**±184.59	**102.04**±25.26	**6.18**±3.32
	Fore-Aft	**738.43**±240.57	**128.30**±24.96	**8.76**±4.81
	Vertical	**390.95**±66.32	**92.38**±14.86	**4.97**±1.94
	LARP	**88.43**±52.85	**12.01**±2.69	**17.71**±26.06
	RALP	**73.96** ±31.38	**10.77**±2.95	**11.25**±5.73
	Yaw	**145.45** ±57.96	**13.49** ±5.15	**24.00**±9.29

**Table 2:** Subject-averaged maximum value, mean, and kurtosis for active everyday activities. The maximum and mean values are expressed in mG for the Lateral, Fore-Aft and Vertical linear acceleration while they are expressed in deg/s for the LARP, RALP and Yaw angular velocity.

		Maximum value	Mean	Kurtosis
Riding a bike	Inter-Aural	**927.42**±303.13	**187.97**±37.13	**5.67**±2.49
	Fore-Aft	**2074.91**±951.18	**339.63**±47.72	**6.89**±3.79
	Vertical	**1890.89**±539.43	**319.32**±53.82	**5.91**±0.77
	LARP	**157.24**±48.85	**28.97**±9.41	**5.87**±0.96
	RALP	**148.78**±48.08	**25.40**±5.22	**6.34**±2.44
	Yaw	**192.07**±65.69	**22.70**±5.91	**19.08**±11.48
Jump up	Inter-Aural	**1000.34**±305.80	**259.78**±70.82	**5.12**±2.06
	Fore-Aft	**2792.71**±716.09	**837.39**±336.31	**4.30**±1.75
	Vertical	**3661.25**±725.20	**1301.96**±452.69	**4.41**±2.73
	LARP	**174.61**±54.01	**54.59**±17.93	**3.83**±0.98
	RALP	**183.80** ±26.37	**51.18**±13.21	**4.76**±1.67
	Yaw	**112.81** ±30.00	**33.62**±9.94	**3.92**±0.67
Hoping on one leg	Inter-Aural	**1351.27** ±399.97	**350.3**±96.87	**5.31**±2.21
	Fore-Aft	**2379.55** ±723.71	**616.84**±178.69	**5.62**±2.65
	Vertical	**3365.23** ±837.46	**1094.35**±230.98	**3.84**±1.44
	LARP	**181.62** ±46.78	**56.36**±26.35	**4.49**±2.46
	RALP	**164.39** ±45.03	**49.37**±19.67	**4.38**±2.12
	Yaw	**146.30** ±54.40	**41.84**±19.39	**4.77**±1.20
Jumping forward	Inter-Aural	**1425.52** ±650.74	**341.84**±166.34	**6.27**±3.00
	Fore-Aft	**4364.85** ±1661.65	**1374.48**±463.03	**4.10**±1.78
	Vertical	**4588.08** ±1879.68	**1213.09** ±423.06	**5.11** ±1.62
	LARP	**240.90** ±76.84	**79.12** ±27.34	**4.10** ±1.44
	RALP	**221.36** ±54.48	**72.09** ±16.57	**4.07** ±2.05
	Yaw	**141.02** ±40.08	**41.83** ±11.53	**4.28** ±2.24
Running on pavement	Inter-Aural	**1278.94** ±378.67	**310.81** ±75.24	**4.04** ±0.82
	Fore-Aft	**1910.25** ±505.52	**391.52** ±95.79	**5.52** ±2.16
	Vertical	**2934.64** ±628.92	**1256.84** ±140.45	**3.20** ±0.56
	LARP	**161.16** ±38.64	**40.61** ±8.57	**3.74** ±0.56
	RALP	**169.03** ±44.36	**39.73** ±7.58	**4.05** ±1.01
	Yaw	**217.69** ±51.27	**36.75** ±9.37	**10.17** ±4.18
Soccer	Inter-Aural	**3511.38** ±1554.58	**407.31** ±79.10	**9.40** ±3.65
	Fore-Aft	**4402.61** ±1894.98	**483.77** ±47.94	**11.48** ±5.08
	Vertical	**3839.55** ±757.27	**523.51** ±108.30	**5.55** ±1.55
	LARP	**370.08** ±127.05	**51.32** ±7.60	**8.16** ±2.37
	RALP	**302.13** ±87.22	**39.59** ±5.41	**8.93** ±2.84
	Yaw	**446.49** ±48.80	**71.97** ±15.01	**8.02** ±1.67
Sprinting	Inter-Aural	**2331.56** ±779.71	**563.48** ±141.95	**5.67** ±2.76
	Fore-Aft	**2492.69** ±574.63	**674.72** ±148.58	**4.18** ±0.85
	Vertical	**4347.48** ±1139.62	**1246.36** ±193.90	**5.63** ±2.58
	LARP	**224.47** ±42.59	**61.54** ±11.98	**4.26** ±1.22
	RALP	**218.67** ±49.06	**60.26** ±11.08	**4.33** ±1.29
	Yaw	**255.02** ±94.36	**54.15** ±13.13	**7.33** ±2.55
Going up the stairs	Inter-Aural	**1034.97** ±400.27	**226.13** ±88.39	**7.49** ±5.96
	Fore-Aft	**1591.46** ±792.19	**362.63** ±93.87	**4.70** ±2.29
	Vertical	**1872.52** ±528.27	**479.74** ±106.25	**4.26** ±1.89
	LARP	**142.97** ±70.68	**35.61** ±13.02	**5.30** ±2.17
	RALP	**151.15** ±25.09	**32.87** ±6.55	**5.53** ±1.70
	Yaw	**278.80** ±132.04	**54.09** ±11.38	**7.84** ±4.85
Walking	Inter-Aural	**400.27** ±358.22	**88.39** ±46.14	**5.96** ±1.36
	Fore-Aft	**1291.00** ±394.71	**237.21** ±61.93	**5.70** ±1.84
	Vertical	**1249.45** ±449.41	**345.55** ±114.49	**5.08** ±1.44
	LARP	**154.09** ±68.26	**24.28** ±9.65	**8.05** ±3.20
	RALP	**128.95** ±39.89	**21.58** ±6.58	**6.74** ±1.96
	Yaw	**310.53** ±114.97	**29.84** ±12.62	**16.73** ±5.86
Running in the Woods	Inter-Aural	**2391.69** ±593.57	**381.68** ±40.16	**6.60** ±1.28
	Fore-Aft	**2664.69** ±679.84	**506.69** ±74.16	**5.98** ±1.78
	Vertical	**4596.69** ±913.79	**862.23** ±64.73	**5.38** ±1.02
	LARP	**265.88** ±59.78	**44.43** ±4.30	**7.41** ±2.59
	RALP	**206.85** ±33.59	**37.61** ±4.39	**5.88** ±1.68
	Yaw	**371.55** ±103.30	**45.49** ±5.41	**15.37** ±5.40

### Voluntary self-motion causes deviation from scale invariance primarily for translational envelope signals

What causes deviation from scale invariance in the envelopes of natural self-motion signals? Previous studies of other sensory modalities have shown that active movement can alter the statistics of natural visual input impinging upon sensors [52]. If this is the case in the vestibular system, then the deviation from scale invariance seen in the envelope of vestibular signals should be due to voluntary movements made during everyday activities (e.g., walking).

**Fig 3:**
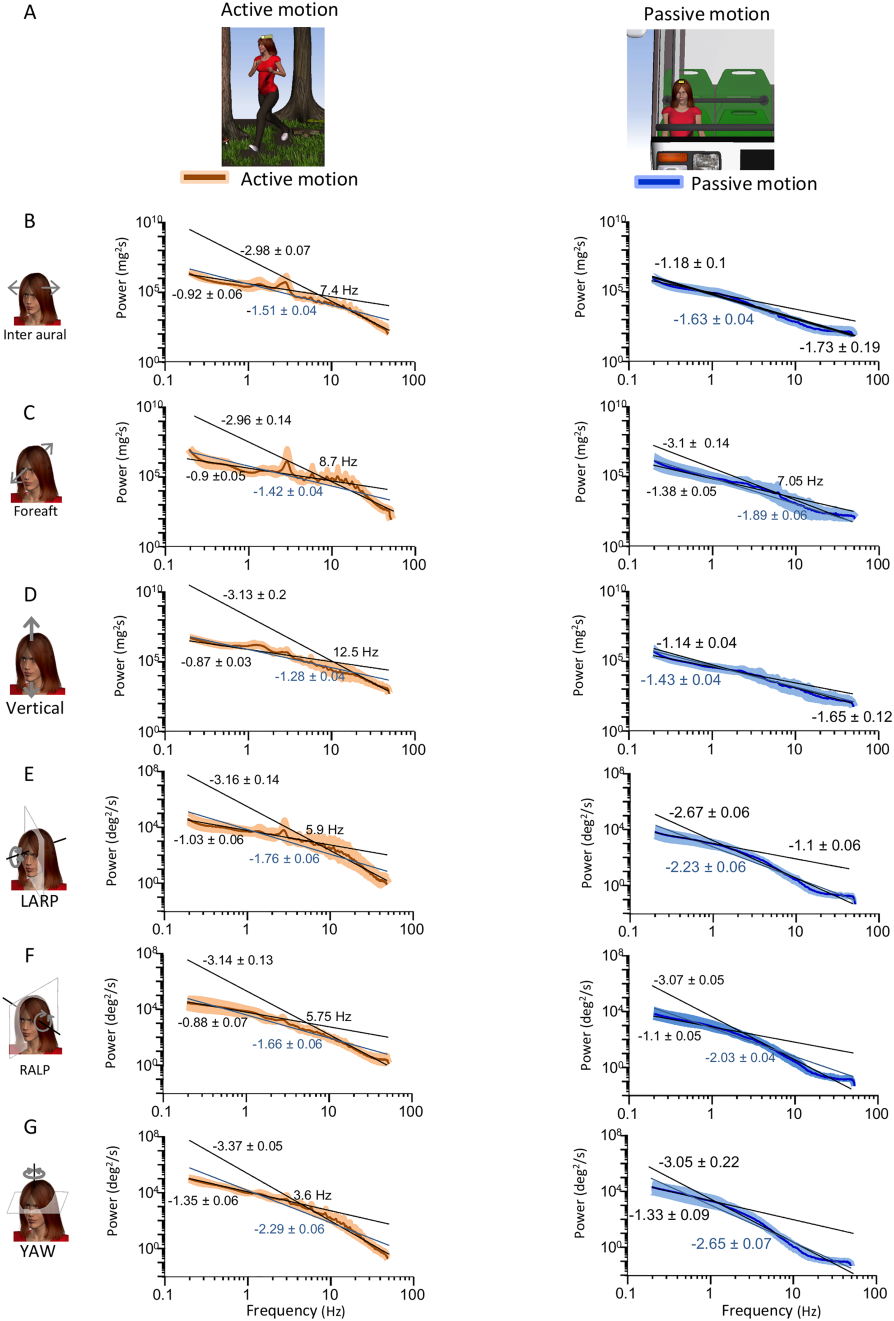
Active motion introduces deviation from scale invariance in the envelopes of natural translational self-motion signals recorded along the Inter-Aural and Vertical axes. **A:** Schematic showing a subject engaged in active self-motion (left) and in passive self-motion (right). **B,C,D,E, F, G:** Subject-averaged envelope power spectra for active (left panels) and passive (right panels) activities for inter aural (**B**), Fore-Aft (**C**), Vertical (**D**), LARP (**E**), RALP (**F**), and YAW (**G**). In each case, the power spectra were fitted using two power laws over the low and high frequency ranges (black lines) as well as by a single power law over the entire frequency range (blue lines). Also shown are the best-fit power law exponents with confidence interval as well as the transition frequency.

**Fig 4:**
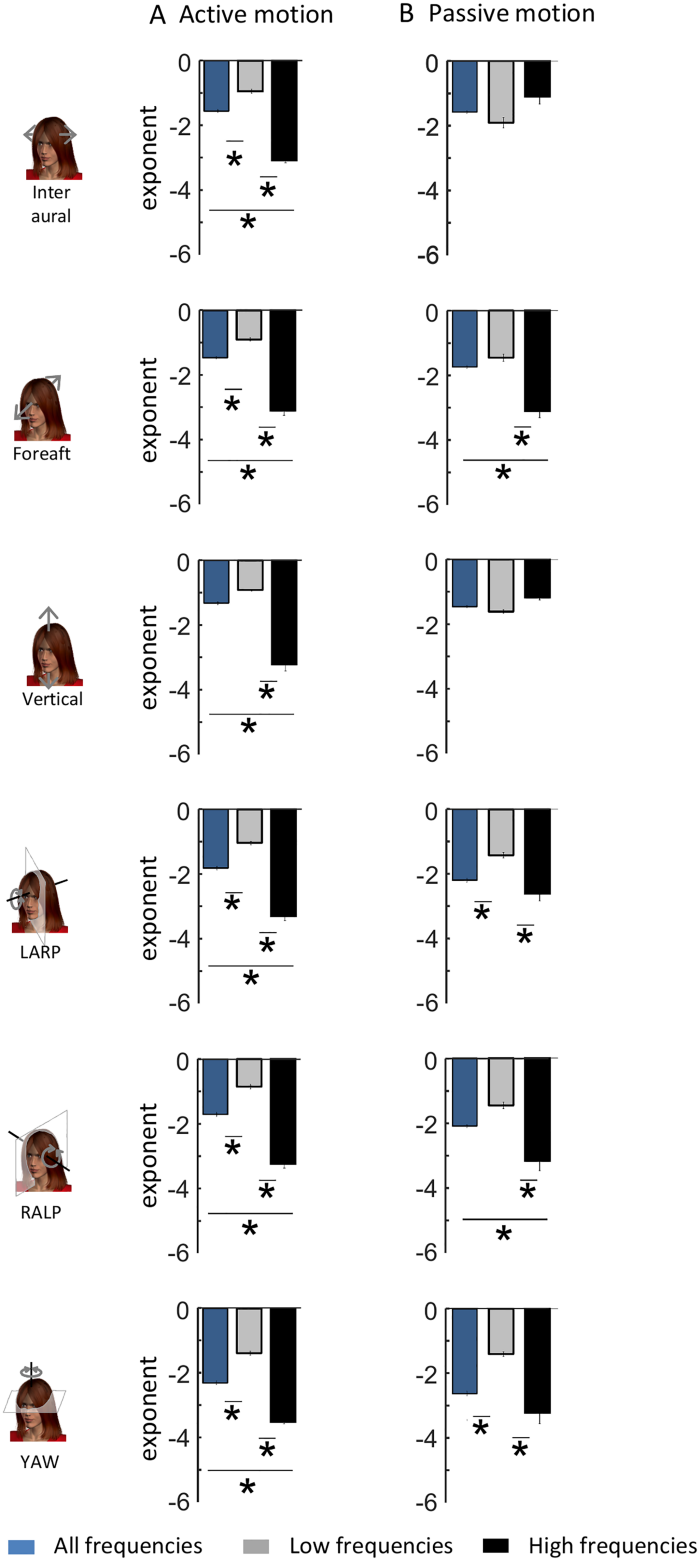
Comparison between the spectral properties of envelope signals recorded during active and passive self-motion. **A:** Subject-averaged best-fit power law exponents over the low (gray) and high (black) frequency ranges for all six motion dimensions for active self-motion. Also shown for comparison are the subject-averaged best-fit power law exponents for a single power law over the entire frequency range (blue). **B:** Subject-averaged best-fit power law exponents over the low (gray) and high (black) frequency ranges for all six motion dimensions for passive self-motion. Also shown for comparison are the subject-averaged best-fit power law exponents for a single power law over the entire frequency range (blue). “*” indicates statistical significance at the p = 0.01 level using a one-way ANOVA.

To test whether active movements contribute to causing deviation from scale invariance, we segregated self-motion signals resulting primarily from active activities from those resulting primarily from passive activities (Fig 3A) and compared the power spectra of their respective envelopes. We found that the envelope power spectra for signals resulting from active motion were qualitatively similar to those obtained across our entire dataset (compare Figs 3B, 3C, 3D, 3E, 3F and 3G to 2A). Indeed, power spectra for signals resulting from active motion decayed more slowly over low frequencies and more sharply over high frequencies (Fig 3B, 3C, 3D, 3E, 3F and 3G, left panels). Consequently, these were well fit by two power laws with different exponents over the low and high frequency ranges (black lines) rather than a single power law over the entire frequency range (blue lines) (Fig 3B, 3C, 3D, 3E, 3F and 3G, left panels). Indeed, the population-averaged best-fit low and high frequency power law exponents were almost always significantly different from one another as well as from the best-fit single power law exponent (Fig 4A).

We next compared the power spectra of envelope signals resulting from passive motion (Fig 3B, 3C, 3D, 3E, 3F and 3G, right panels) to those of envelope signals resulting from active motion (Fig 3B, 3C, 3D, 3E, 3F and 3G, left panels). For Inter-Aural and Vertical translations, we found that the power spectra resulting from passive activities tended to decay more uniformly as a function of increasing frequency than those from active activities (Fig 3B and 3D, compare left and right panels). Although these spectra could also be well fit by two power laws over the low and high frequency ranges, the low and high frequency best-fit power law exponents obtained by using a two power law model were similar to one another in value. Indeed, further analysis revealed that the two best-fit power law exponents were not significantly different from one another, or from the exponent obtained by fitting a single power law over the entire frequency range (Fig 4B). These results suggest that active movements strongly contribute to causing deviation from scale invariance for translational envelope signals along these axes.

For rotations (i.e., LARP, RALP, and YAW) as well as Fore-Aft translations, we found that the envelope power spectra of signals resulting from active and passive activities were more similar in structure as they both decayed more slowly for low frequencies and more sharply for high frequencies (Fig 3C, 3E, 3F and 3G, compare left and right panels). Consequently, the envelope spectra of rotational signals resulting from passive activities were better fit by two power laws with different exponents over the low and high frequency ranges than by a single power law over the entire frequency range. Further analysis revealed that the two best-fit power law exponents were for the most part significantly different from one another as well as from the exponent obtained by fitting a single power law over the entire frequency range (Fig 4B). Our results thus suggest that active self-motion at best contributes minimally to causing deviation from scale invariance for rotational self-motion envelopes as well as Fore-Aft translations.

### Filtering by the human body gives rise to deviation from scale invariance primarily for rotational self-motion

We next investigated whether filtering by the human body could contribute to causing deviation from scale invariance in envelope self-motion signals. This is because previous studies have shown that such filtering causes deviation from scale invariance for carrier self-motion signals [40]. Indeed, vestibular signals experienced during typical everyday activities are transmitted through the body before reaching the vestibular sensors in the head. For example, when a person is riding in a vehicle, vibrations from the ground travel through the subject’s body prior to reaching the head. Similarly, filtering by the human body will also be present during active self-motion (e.g., vibrations caused by the foot striking the ground during walking travel through the subject’s body prior to reaching the head).

**Fig 5:**
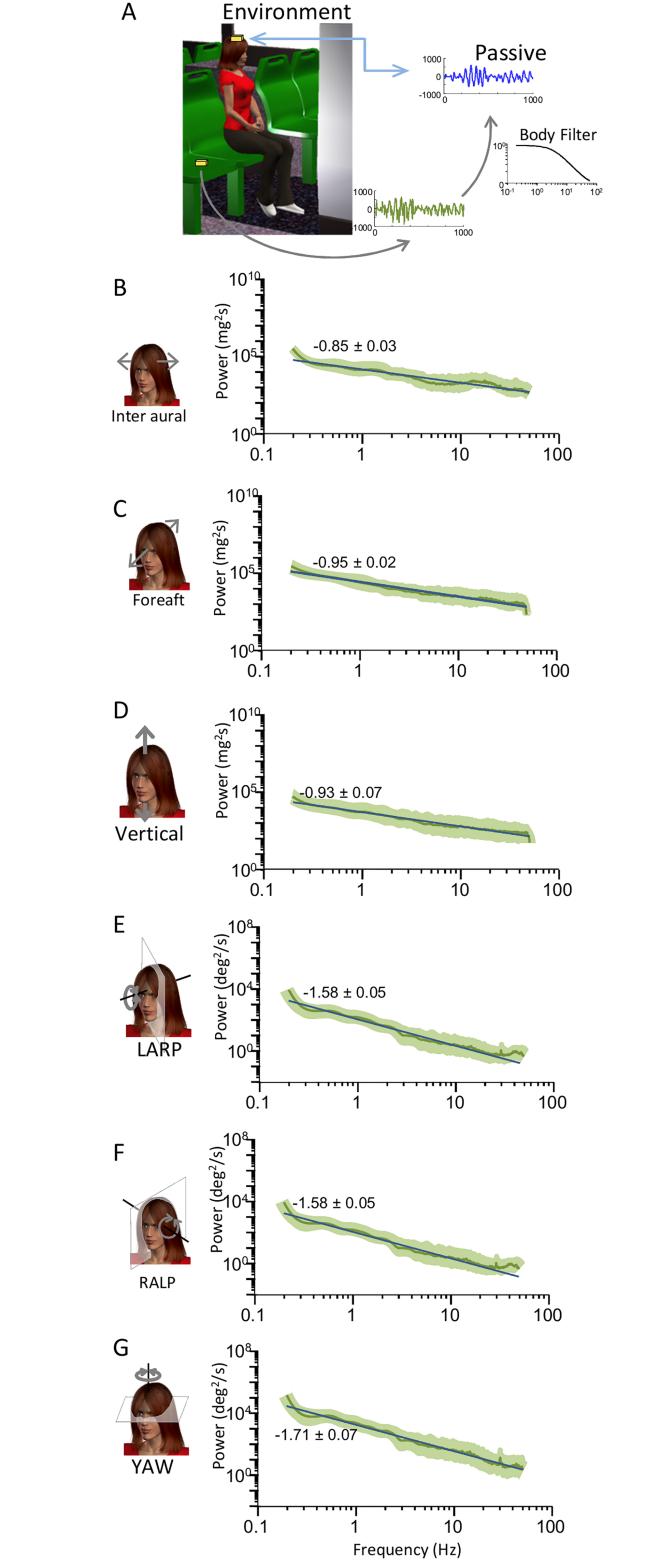
Statistics of environmental signals obtained when the subject is absent. **A:** Schematic showing the MEMS module (gold box) located on the subject’s head and placed on the seat during passive self-motion. **B,C,D,E, F, G:** Trial-averaged power spectra of signals in the external environment (green) during passive self-motion for inter aural (**B**), Fore-Aft (**C**), Vertical (**D**), LARP (**E**), RALP (**F**), and YAW (**G**). The power spectra were in general well fit by a single power law over the entire frequency range (blue lines).

**Fig 6:**
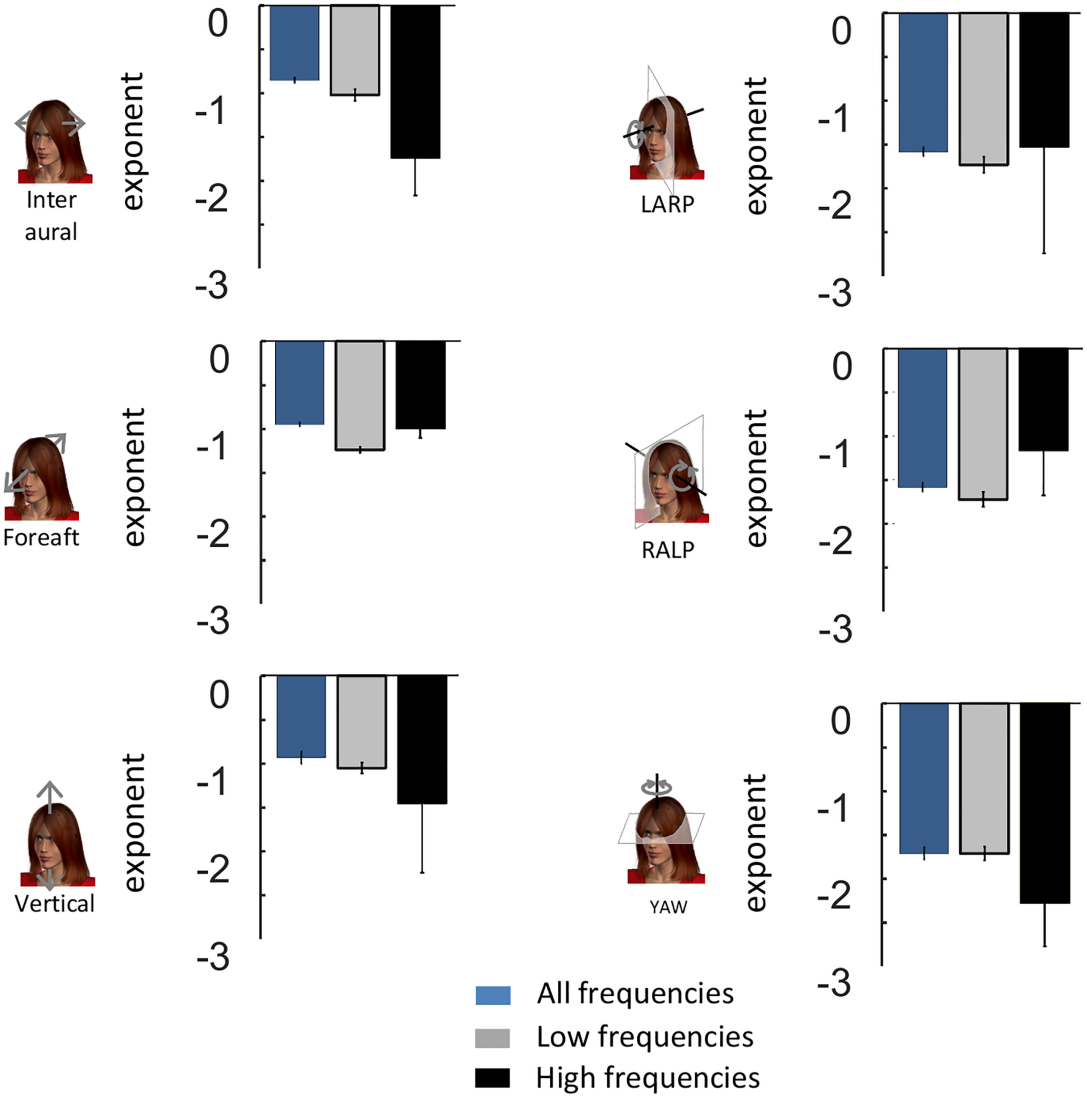
External envelope signals display scale invariance. Subject-averaged best-fit power law exponents for the envelopes of external stimuli during passive self-motion when fitting a power law over the entire frequency range (blue) and when fitting two power laws over the low (gray) and high (black) frequency ranges.

To test whether filtering by the human body contributes to causing deviation from scale invariance for natural self-motion envelopes, we compared envelope signals obtained during passive self-motion measured at the subject’s head to those measured when the subject is absent (i.e. external stimuli) (Fig 5A). Specifically, we investigated the contributions of filtering by the human body during passive self-motion in order to distinguish them from the potential effects of active self-motion. Our results show that, overall, the power spectra of self-motion envelope signals measured externally were well-fit by a single power law over the entire frequency range across all six motion dimensions (Fig 5B, 5C, 5D, 5E, 5F and 5G). Indeed, the low and high frequency best-fit power law exponents were not significantly different from one another or from the one obtained by fitting a single power law over the entire frequency range (Fig 6). We note that the power spectra of external stimuli are lower than that measured when the subject is present (compare curves in Figs 3 and 5). These differences are likely due to resonance properties of the human body (see e.g. [53]) whose frequency highly depends on posture (see e.g. [54]).

When considering rotational and Fore-Aft translational envelope signals, the power spectra of signals measured at the subject’s head during passive self-motion decayed slowly for low and more sharply for high frequencies (Fig 3C, 3E, 3F and 3G, right panels) whereas those measured when the subject is absent instead decayed uniformly (Fig 5C, 5E, 5F and 5G). These results suggest that filtering by the human body causes significant deviation from scale invariance for rotational envelope signals and Fore-Aft translations. However, when instead considering Inter-Aural and Vertical translations, the power spectra of envelope signals measured at the subject’s head (Fig 3B and 3D, right panels) and when the subject is absent (Fig 5B and 5D) all tended to decay uniformly with increasing frequency. These results suggest that filtering by the human body causes minimal deviation from scale invariance for Inter-Aural and Vertical translational envelope signals.

Thus, our results suggest that translational and rotational envelope signals deviate from scale invariance primarily for different reasons. Specifically, while active self-motion makes the primary contribution for the former, filtering by the body instead makes the primary contribution for the latter. The one notable exception to this rule is Fore-Aft translations for which filtering by the human body rather than active self-motion causes deviation from scale invariance.

### Predicting afferent responses to natural envelopes

**Fig 7:**
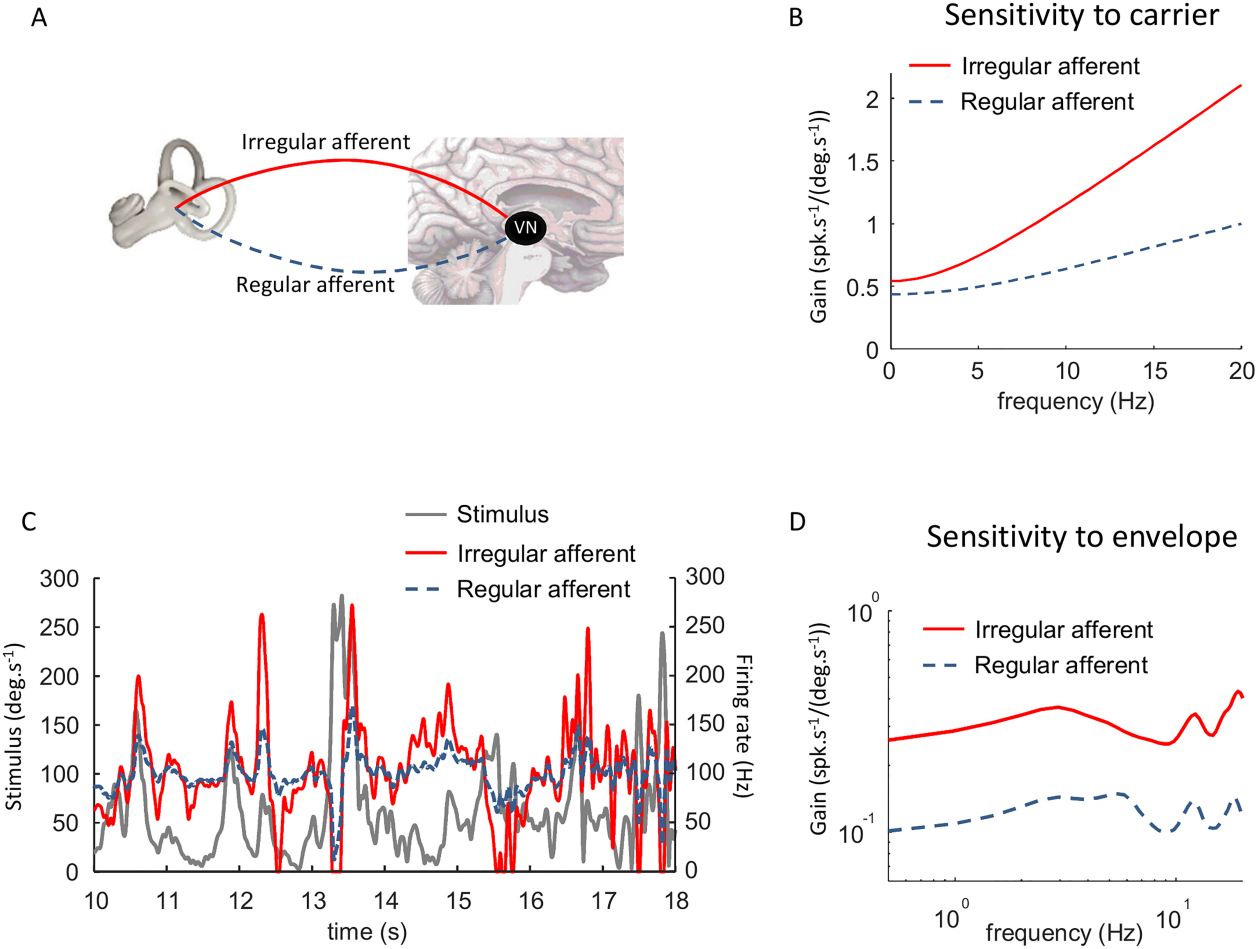
Well-established models of the vestibular periphery predict that irregular afferents have greater sensitivities to envelopes than their regular counterparts. **A:** Schematic showing the vestibular end organs as well as regular and irregular vestibular afferents projecting to the vestibular nuclei. **B:** Sensitivity to the carrier for the regular (dashed black) and irregular (solid red) model afferents. **C:** Time series showing a segment of the envelope stimulus (solid black) and the responses of the model regular (dashed black) and irregular (solid red) afferents. **D:** Gain to the envelope as a function of frequency for the regular (dashed black) and irregular (solid red) model afferents. In both cases the gain is relatively independent of frequency but is about twice higher for the irregular model afferent.

So far we have focused on characterizing the statistics of natural self-motion envelopes as well as potential mechanisms that cause deviation from scale invariance in their structure. In the following, we instead focus on making predictions as to how peripheral vestibular afferents respond to natural self-motion envelopes. To do so, we used well-established models that reproduce the response dynamics of afferents seen experimentally (Fig 7A, see Methods). Specifically, we first used transfer functions based on experimental findings [34] to predict the firing rate response to the carrier signal. Importantly, the sensitivity of the model irregular afferent to the carrier was higher than that of its regular counterpart across the relevant frequency range [28, 55] (Fig 7B). Fig 7C shows the predicted responses of the model regular and irregular afferents to a natural stimulus. Notably, the stimulus gave rise to greater changes in firing rate for the model irregular afferent because of its higher sensitivity. As such, the model irregular afferent tends to be driven more into cutoff (i.e. cessation of activity) and saturation than its regular counterpart (Fig 7C). In order to quantify tuning to the envelope, we computed the sensitivity as a function of temporal frequency (see Methods). This is a standard measure that has been used previously to characterize neural responses to envelopes in the electrosensory system [8, 10, 21] and that is equivalent to temporal modulation transfer function measures that have been used extensively to characterize neural responses to envelopes in the auditory system [48, 49] (see [14] for review). Fig 7D shows the envelope sensitivity as a function of frequency for both the model regular and irregular afferents in response to the envelope. Both were relatively independent of envelope frequency but the envelope sensitivity computed for the model irregular afferent was approximately twice that computed for the model regular afferents. Thus, our simulations predict that irregular afferents will display higher sensitivities to envelopes than their regular counterparts.

## Discussion

### Summary of results

We investigated the envelope statistics of self-motion stimuli experienced by human subjects during everyday activities. We found that these could reach high values (~450deg/s for rotations and ~4G for translations), were characterized by probability distributions with high kurtosis, and displayed power spectra that decreased slowly for lower (< 2 Hz) and more steeply at higher (> 2 Hz) frequencies. These statistics were seen across all six motion dimensions. We found that different mechanisms underlie deviation from scale invariance depending on whether one considers translational or rotational self-motion envelopes. Indeed, our data suggests that active self-motion and filtering by the human body make the primary contribution to deviation from scale invariance for the former and latter, respectively. The one notable exception to this rule is Fore-Aft translations, for which filtering causes deviation from scale invariance. To understand the implications of the present findings for envelope coding by the vestibular system, we used well-established models of the vestibular periphery to simulate afferent responses to natural envelope stimuli. Our simulations predict that irregular afferents are more sensitive to envelopes than their regular counterparts.

### Functional roles of envelopes in vestibular pathways

Envelopes can carry behaviorally relevant information. For example, in the visual system, these are crucial for edge detection in visual scenes [56, 57] while, in the auditory system, they carry crucial information required to perceive timbre in music as well as speech perception [14, 22, 23]. In the active electric sense of weakly electric fish, envelopes carry crucial information about both distance and identity of conspecifics [19, 20]. While previous studies carried out in other systems have shown that natural envelope signals display scale invariance [18], our results suggest that natural envelope self-motion signals instead display deviation from scale invariance due to active self-motion and filtering by the human body. This is interesting, since studies of natural stimuli have typically looked at the stimuli themselves (e.g., natural visual images) without taking into account active movements (e.g., eye saccades when freely viewing an image). Indeed, a recent study has shown that active eye movements cause deviation from scale invariance in natural first-order visual signals [52]. It is thus conceivable that active motion will also cause deviation from scale invariance for second-order (i.e. envelope) sensory signals in other systems.

While the functional role of envelopes has not been fully established in the vestibular system, there is evidence that their detailed structure is processed and retained in vestibular pathways. We speculate that envelope coding is important for central processes that integrate vestibular input over time to adapt to the current amplitude range of self-motion stimuli. Indeed, there is evidence that vestibular reflexive and perceptual responses to a sustained directional stimulus are reduced over time [38, 39], and that vestibular perceptual and balance responses are regulated, over the course of minutes, as a function of the self-motion envelope [37]. Furthermore, psychophysical studies in humans have suggested that a mechanism for inducing motion sickness involves integrating the amplitude of vibrations over time [58]. The regulation of amplitude range, reciprocal connections between the vestibular cerebellum (i.e., cerebellar nodulus and uvula) and vestibular nuclei are known to lengthen the time constant of the semicircular canals. This process, termed velocity storage, shapes the dynamics of both the perception of self-motion and vestibular-driven behaviors. Notably, motion sickness sensitivity is decreased following training that reduces velocity storage [59–65], providing further support for the proposal that motion sickness is triggered by the integration of motion stimuli over time. Moreover, anti-motion sickness drugs enhance adaptation of this mechanism allowing progressive exposure to higher levels of stimulation without symptoms being elicited [66–68]. Interestingly, alterations of velocity storage may also contribute to vertigo susceptibility in vestibular migraine patients [69, 70], suggesting that the envelopes of vestibular signals have additional clinical relevance.

### Envelope coding in vestibular pathways: Functional role of neuronal variability

It is well-known that vestibular afferents display strong heterogeneities in their responses to self-motion stimulation that are in part due to differential hair cell morphology and patterns of innervation. These neurons are typically classified as either regular or irregular based on their resting discharge variability [28]. Despite over 40 years of work, the functional role of each afferent class is still not fully understood.

As stated above, the envelope of a signal can only be extracted mathematically by performing a nonlinear transformation. The conventional wisdom is that early vestibular processing is inherently linear [28, 32, 33, 71]. However, the stimuli used in these previous studies consisted of artificial sinusoidal and noise stimuli whose amplitude is actually much lower than that seen in natural self-motion [34, 40]. More recent studies have shown that semi-circular and otolith afferents as well as central vestibular neurons display strong nonlinearities in their responses to naturalistic signals [34, 35]. However, static nonlinearities such as rectification and saturation, which are necessary for a neuron to encode second-order attributes [14, 19], tend to be more reliably elicited for irregular afferents experimentally as these tend to have higher sensitivities to carrier self-motion signals than their regular counterparts [34]. Such nonlinearities are necessary in order for neurons to respond to envelopes [14, 19] and our simulations predict that they will give rise to envelope responses in vestibular afferents that will be transmitted to higher order brain areas. Moreover, our simulations predict that regular and irregular afferents have different functional roles for envelope coding. If correct, this would provide new insight into the longstanding problem of why the primate vestibular system has two afferent classes. Further studies are however needed to verify these predictions and, if true, characterize the tuning properties of individual regular and irregular afferents, as well as those of central vestibular neurons, to envelopes.

### Comparison between the statistics and coding of carrier and envelope self-motion signals

Our results show that active movements cause deviations from scale invariance for translational self-motion envelope signals prior to sensory transduction. As such, our results strongly differ from those of a recent study that instead investigated the statistics of carrier self-motion signals [40]. Indeed, this prior study reported that filtering by the body is primarily responsible for deviations from scale invariance in both translational and rotational carrier self-motion signals prior to reaching the vestibular sensors in the head [40]. Thus, the mechanisms that cause deviation from scale invariance in carrier and envelope self-motion signals are different when considering Inter-Aural and Vertical translations and similar when instead considering rotations and Fore-Aft translations.

This has important implications for neural coding as there is growing evidence that sensory systems can efficiently process natural stimuli by ensuring that coding strategies are matched to input statistics [1–5, 11, 72]. While the statistics of natural stimuli in other sensory modalities (e.g. auditory, visual) have been known for quite some time [12, 73], the statistics of natural self-motion stimuli have only been investigated recently in humans [40] and non-human primates [34]. Importantly, a recent study has shown that irregular semicircular and otolith vestibular afferents can more efficiently encode natural carrier self-motion signals than their regular counterparts, suggesting that the coding strategies used by the primate vestibular system are adapted to natural carrier self-motion statistics [34]. We speculate that the probability distributions of envelope signals presented in the current study, together with the tuning properties of afferents to envelopes, might show that irregular afferents are more adapted to natural envelope statistics than their regular counterparts. Moreover, we predict that, if vestibular coding strategies are matched to natural self-motion statistics, then our results showing that translational envelopes resulting from active and passive self-motion have fundamentally different statistics implies that these should be processed differentially in the brain. Further experimental studies are however needed to test these predictions.

### Parallel processing of carrier and envelope signals

The coding of both carrier and envelope components of natural stimuli remains an important problem in systems neuroscience. While the statistics of carrier vestibular signals have been recently reported [40], the statistics of envelope vestibular signals had not been investigated prior to this study. Our results characterizing the statistics of natural envelope vestibular signals pave the way for future electrophysiological investigations aimed at understanding how these signals are processed in the brain. To that effect, a general strategy used by the brain to encode both components is to devote separate neural circuits in order to encode each. Indeed, such parallel processing is thought to occur in the visual system [56, 57, 74] and has been demonstrated in the electrosensory system [45]. Based on arguments presented above, it is possible that such parallel processing might begin to occur as early as the vestibular periphery since irregular afferents are predicted to respond more strongly to envelope self-motion signals than their regular counterparts. However, how central vestibular neurons integrate input from both afferent classes in order to ensure that both carrier and envelope components are accurately represented is not clear and should be the focus of future studies.

## Supporting information

S1 File This file contains the data used to generate the figures. (ZIP)
